# Newly Discovered Mechanism for Chlorpyrifos Effects on Neurodevelopment

**DOI:** 10.1289/ehp.120-a270a

**Published:** 2012-07-02

**Authors:** Carol Potera

**Affiliations:** Carol Potera, based in Montana, has written for *EHP* since 1996. She also writes for *Microbe*, *Genetic Engineering News*, and the *American Journal of Nursing*.

The organophosphate insecticide chlorpyrifos (CPF) causes loss of brain cells in young rats,^1^ and prenatal CPF exposure in humans has been linked to a significant reduction in childhood IQ scores.^2^ Now a non-invasive magnetic resonance imaging (MRI) study reveals that in children exposed to the pesticide *in utero*, CPF alters the structure of brain regions that govern a broad range of behavioral outcomes, offering new insight into the way in which CPF affects the central nervous system.^3^

CPF was once used for food crops, golf courses, indoor pest control, and pet collars.^4^ Residential use was phased out in 2001 after the U.S. Environmental Protection Agency (EPA) released a revised risk assessment of the pesticide calling for stronger protection for children and workers.^5^ However, CPF is still widely used in agriculture, potentially exposing farm workers and people who live close to farms. Eating grains, fruits, vegetables, and other foods containing CPF residues also can expose people to very low levels. In the most recent U.S. Department of Agriculture Pesticide Data Program survey, levels of CPF detected in produce were 3–500 times lower than EPA tolerance levels for individual foods.^6^

The current study is part of a larger ongoing cohort study of minority women and their children conducted by the Columbia Center for Children’s Environmental Health (CCCEH) at Columbia University. The project started in 1997 before residential use of CPF was phased out. CPF measured in the children’s umbilical cords indicated the degree to which they had been exposed to the pesticide *in utero*, largely through their mothers’ exposures to pesticides sprayed in apartment buildings.^7^

Previous reports from CCCEH investigators found an association between higher CPF exposures and lower birth weights.^8^ By age 3 years, children with higher *in utero* CPF exposure were more likely than lower-exposure children to score lower on tests of cognitive and psychomotor development,^9^ and by age 7 they were more likely to have lower IQ and working memory scores.^2^ The current study assessed MRI data for 40 children at age 6–11 years, half of whom had umbilical plasma CPF levels of 4.39 pg/g or greater and half of whom had levels below 4.39 pg/g. Exposure to lead, another environmental toxicant linked to cognitive problems, was low in all the children.

Children in the higher-exposed group were more likely to have significant enlargement of the regions of the brain that control attention, language, social cognition (e.g., ability to recognize faces), emotion and inhibition, and executive functions (e.g., planning and reasoning), compared with the lower-exposed group.^3^ These differences are consistent with findings from animal studies of CPF exposure.^1^ In the lower-exposed group, children with greater brain enlargement tended to have lower IQ scores, but this relationship was not seen in the higher-exposed group.^3^ “Bigger is not always better when it comes to the brain,” says coauthor Bradley Peterson, a psychiatrist at Columbia University who performed the MRIs.

The scans revealed that much of the brain enlargement consisted of glia, or white matter.^3^ CPF damages neurons and glia and generates scar tissue in animal models,^1^ and Peterson speculates similar damage could account for the enlargement seen in the children. Higher CPF exposure also was associated with reduction or reversal of normal sex-related differences in brain development. For instance, the right parietal lobe is generally larger in girls than boys, but this was reversed in higher-exposed children. The behavioral consequences of these alterations, if any, are unknown.^3^

Brain differences between the two groups were found at CPF exposure levels well below current EPA dietary reference doses (0.005 mg/kg/day for the general public and 0.0005 mg/kg/day for women and children).^5^ This standard represents the lowest level at which CPF exposure is associated with inhibition of the enzyme acetylcholinesterase, which breaks down the neurotransmitter acetylcholine. An excess of acetylcholine can cause dizziness, tremors, and other neurologic symptoms.^4^

**Figure:**
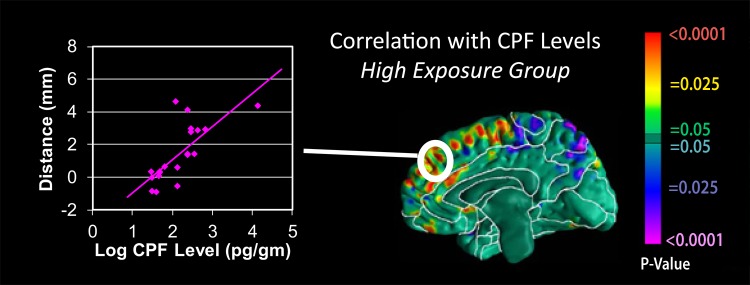
Warm colors indicate positive correlations between enlargement of the cerebral surface and CPF exposure. Cool colors indicate inverse correlations.

“Until now, the guidelines for determining acceptable exposure to this pesticide were based on the assumption that the central nervous system effects of chlorpyrifos were due to dysregulation of neurotransmission,” explains Joseph Jacobson, a psychiatry professor at Wayne State University School of Medicine in Detroit, Michigan. He says the new MRI data “strongly suggest a very different mechanism—one that acts at much lower levels of exposure than were previously believed to be associated with increased risk.” Therefore, he adds, this study suggests that the EPA guidelines for CPF exposure may need to be revised.

Study coauthor Robin Whyatt, deputy director of the CCCEH, agrees. “Our data and other experimental studies show that it’s highly likely that chlorpyrifos affects fetal development by mechanisms other than acetylcholinesterase inhibition and at doses lower than those that inhibit acetylcholinesterase,” she says.

The EPA is currently finalizing a preliminary risk assessment for CPF that was issued for public comment in summer 2011 as part of the periodic re-evaluation of pesticides registered for use in the United States.^10^ On 10–13 April 2012 the Federal Insecticide, Fungicide, and Rodenticide Act Scientific Advisory Panel met to review the newest information about health issues related to CPF exposure. The MRI study “will be evaluated, along with all the other data the agency is assessing, as it works to finalize the human health risk assessment,” according to an EPA spokesman who spoke on condition of anonymity.

The combination of epidemiologic and brain imaging data in this study provides some of the strongest evidence to date that prenatal exposure to CPF causes neurodevelopmental problems in children, according to Peterson. He says this type of protocol “should become the wave of the future for looking at how environmental [toxicants] contribute to neuropsychiatric illness in children.”
